# Quantifying the speech-gesture relation with massive multimodal datasets: Informativity in time expressions

**DOI:** 10.1371/journal.pone.0233892

**Published:** 2020-06-02

**Authors:** Cristóbal Pagán Cánovas, Javier Valenzuela, Daniel Alcaraz Carrión, Inés Olza, Michael Ramscar

**Affiliations:** 1 Department of English Philology, University of Murcia, Murcia, Spain; 2 Department of Quantitative Linguistics, Eberhard Karls University of Tübingen, Tübingen, Baden-Württemberg, Germany; 3 Institute for Culture and Society, University of Navarra, Pamplona, Navarra, Spain; University of Birmingham, UNITED KINGDOM

## Abstract

The development of large-scale corpora has led to a quantum leap in our understanding of speech in recent years. By contrast, the analysis of massive datasets has so far had a limited impact on the study of gesture and other visual communicative behaviors. We utilized the UCLA-Red Hen Lab multi-billion-word repository of video recordings, all of them showing communicative behavior that was not elicited in a lab, to quantify speech-gesture co-occurrence frequency for a subset of linguistic expressions in American English. First, we objectively establish a systematic relationship in the high degree of co-occurrence between gesture and speech in our subset of expressions, which consists of temporal phrases. Second, we show that there is a systematic alignment between the informativity of co-speech gestures and that of the verbal expressions with which they co-occur. By exposing deep, systematic relations between the modalities of gesture and speech, our results pave the way for the data-driven integration of multimodal behavior into our understanding of human communication.

## Introduction

Among the multiple acoustic and visual features that can be part of the communicative signal in everyday face-to-face situations, language stands out as the most deeply structured. Other modalities, such as gesture or gaze, although also showing structure, may seem less patterned, often providing information that is merely complementary to the linguistic message. But this impression is not entirely based on empirical evidence. In fact, we still have a very limited understanding of how systematic the interplay might be between verbal and non-verbal cues in communication. This becomes all the more apparent when examining the expression of specific meanings or functions, which may be connected to concrete forms in language. There is simply so much we do not know about how multimodal information comes together to signal for something in particular. Could multimodal features also be part of a linguistic pattern or grammatical construction [1–9]? Can non-verbal modalities be pervasively and deeply structured in a way that compares with language? Is it adequate to envisage a modular model of communication, involving separate, autonomous semiotic channels, such as speech versus gesture, independent from one another although with various degrees of coordination and overlap? Or is it better to view communication as one single dynamic system, in which all modalities are deeply interdependent and integrated into a unitary signal?

Such questions point at the central problems of segregation and binding in human communication, that is, how participants know which perceptual features from different modalities must be selected for integration and processing as related cues to a certain meaning—e.g. a gesture simulating a timeline alongside a temporal phrase—and what to discard or downplay, such as a hand reaching out for a glass of water while an unrelated verbal expression is being uttered [10]. In the present study, we sought to investigate whether the co-occurrence of language and gesture is systematic for the expression of specific meanings, and whether that systematicity could be driven at least in part by communicative factors, in this case the informativity of the signal as a facilitator of prediction and uncertainty reduction.

To analyze how different modalities are integrated in communication, large-scale quantitative studies are needed. However, data-based research on human multimodal communication must necessarily face a major challenge: the non-verbal modalities, especially the visual ones, are much harder to quantify than speech or text. Indeed, insights in language engineering have taken spoken interfaces from the stuff of science fiction to everyday ubiquity in little over a decade, thanks to the development of massive datasets of speech and text, alongside statistical techniques for analyzing them [11]. By comparison, the ‘unreasonable effectiveness of data’ [12] has not yet made a significant impact on the study of the communicative contributions of other modalities, especially the visual ones [10]. An obvious reason for this is that quantification becomes increasingly–indeed, almost exponentially—complicated as research progresses from text, then to speech, and finally to the full range of behaviors employed in human communication [10]. Text is by nature discrete, and easily quantifiable. Speech then adds the complexity of segmenting a continuous and highly variable signal into quantifiable units, many of which will only be implicitly present in what is actually ‘said’ [13]. But everyday spoken communication involves far more than just ‘words.’ It has been proposed that, throughout a long phylogenetic evolution, at least partially shared with other species, human beings have developed a multimodal communicative system [14] that interconnects a wide range of modalities: non-verbal sounds, rhythm, pace, facial expression, bodily posture, gaze, or gesture, among others. In this diachronic perspective, language is just the ‘tip of the iceberg;’ not just the latest development, but also one that builds on an already rich and complex cognitive and sensorimotor architecture, which already allowed for the nuanced manipulation of the multimodal signal before language appeared.

Of all these non-verbal modalities, gesture has probably received the most attention so far, both independently and in conjunction with other communicative behaviors, in particular with speech. The most recent theories tend to view gesturing as an inextricable part of human communicative behavior [15,16]. Some current proposals link, for example, gestural information to prosody, or gaze-following abilities to the construction of joint attentional frames, alongside other connections between various aspects of language or communication and traits of bodily expression [17–19]. Evidence suggests that speech and gesture are sides of the same cognitive process [20]. Impeding gestures affects speech production, and stutterers also stutter when gesturing [21]. Speakers not only gesture when communicating with other interlocutors, but also when the addressee is not present or cannot see them [22,23].

A powerful initial indicator of a systematic relation between language and gesture would be provided by measuring the frequency of co-occurrence of specific verbal patterns with structurally-related co-speech gesture. This would connect both parts of the signal to the same semantic or communicative functions. However, given that so far it has been challenging to quantify, gesture-speech frequency of co-occurrence has not been the focus of much attention. To date, evidence on speech-gesture frequency of co-occurrence has been provided mainly as a collateral finding in studies dealing with overarching communicative, social, and cognitive factors that condition gestural patterns, and using at most a few dozen instances of gesture [24–27]. In these studies, gestures are typically elicited in experimental settings or during fieldwork, often by asking participants to retell a story based on a written or visual input [28]. Though this methodology presents advantages [29], such as the possibility of event-by-event comparison among speakers, it does not allow for large-scale quantitative analysis of the language-gesture relation, simply because the massively skewed nature of linguistic distributions [30,31] guarantees that specific phrases or sets of phrases will tend to occur at extremely low average frequencies across small sets of participants. Accordingly, many quantitative studies have resorted to counting the overall number of gestures that individual speakers make, without tying them to specific linguistic expressions [32].

Overall, the results of these qualitative and relatively small-scale quantitative studies suggest that there are recurrent patterns in gesture, and that at least some gestural and verbal patterns co-occur systematically. It has recently been suggested that this systematicity facilitates the production and processing of the communicative signal, which has been shown to be interpreted faster and more accurately when it contains multimodal rather than solely unimodal information [10]. However, any claim on the systematicity of the language-gesture relation, or of any other modalities, has yet to be tested by large-scale, quantitative studies of multimodal corpora. Although our knowledge of the matter remains incipient, recently there have been some significant efforts to address the topic of gesture-language co-occurrence through larger quantitative studies, using UCLA’s NewsScape Library of Television News, the same television archive that provided the data for the present study. Analyzing corpora including 200–250 clips, sometimes more, with utterances of the same grammatical constructions [from X to Y, all the way from X PREP Y] these studies have established that there might be very high rates of co-occurrence between specific phrases and gestural patterns, sometimes reaching 80% [33,34]. A co-speech gesture rate of 58% has also been found in constructions headed by aspectualized verbs (e.g. *continue to go*, *stop talking*], with features such as gesture timing, movement, and stroke probably being systematically used to represent different aspectual conceptualizations [35].

Seeking to increase the quantitative power of such studies, as well as to overcome the limitations of studies not using multimodal corpora, we conducted a sizeable study of speech-gesture frequency of co-occurrence across a specific subset of linguistic expressions, in authentic communicative settings. From the same massive dataset of TV News used by the studies just mentioned, we extracted a corpus of over 8,000 videos where speakers were uttering semantically-related verbal patterns, in this case a representative set of conventional time expressions.

We chose temporal expressions because the spatialization of time is a paradigmatic case study of relations between concepts [36,37], and substantial research on gestures co-occurring with speech about time already exists [38–41]. While this work largely aimed at describing underlying representations, it provides characterizations of gesture in this domain that offer a good foundation for our attempt to quantify the relation between gestural and verbal signals.

Looking for factors that could explain the systematic co-occurrence of speech and gesture in relation with specific meanings, we turned to the informativity of the time phrases as a measure that could account for a significant portion of the data. Our hypothesis was that, given a unitary, integrated communicative system that strives to maximize the efficiency of its signals, the combination of modalities would tend to facilitate processing by adding information whenever that is needed to increase the predictability of the message. Therefore, a less predictable structure in one of the modalities, such as a less frequent temporal phrase, would increase the pressure in the system to supply information through other modalities, gesture in this case.

This investigation into the influence of informativity in gesture frequency integrates a theoretical interest with recent methodological developments. The availability of large corpora of speech and text has led to a growth in interest in the quantitative evaluation of the function and efficiency of communicative codes using information theoretic techniques [42]. Information theory characterizes communication as a process in which a message is selected at the information source, encoded into a signal, and transmitted through a physical medium to a receiver, which then decodes the signal to recover the intended message. Coding thus serves the function of facilitating the recovery of transmitted messages, and its efficiency is usually defined as minimizing the average lengths of signals while maximizing rates of communicative success, such that efficient codes make the signals for frequently-used messages shorter than those for infrequent messages. From this perspective, it follows that since gestures involve effort, then if we suppose that they play a role in communication, we should expect that the likelihood that a gesture occurs as part of a signal is a function of the predictability of the message that is being communicated in context.

However, a problem that studies of human communication face is that whereas information systems operate using well defined source codes, the exact information structure of human communicative codes remains a matter of conjecture, as is evidenced by the fact that the role played by gesture in the code—or indeed, whether it plays a role at all—has yet to be quantitatively established [10]. This means that the predictability of linguistic messages can only be estimated at best. In this case, the frequency of the temporal phrases studied in COCA, a large corpus of contemporary American English [43], was used as an estimate of the informativity of the temporal messages that they signal.

It is important to note that although we operationalize predictability in terms of frequency in our analyses, we do not imply that the former can simply be reduced to the latter. It is clear that the structural and distributional properties of languages have evolved to support efficient communication at numerous levels of abstraction [42,44,45], and that notwithstanding the strong correlations typically observed between frequency and the behavioral measures used to asses the predictability of processing, people’s performance on these measures is in fact often better explained by these other properties [46]. However, the fact that frequency does correlate so well with the other factors that modulate the predictability of linguistic processing means that it can serve as a reasonable and–importantly–readily quantified proxy for them, for current purposes.

## Materials and methods

### Corpus building and dataset selection

Data for the study were extracted from the NewsScape Library of Television News (http://newsscape.library.ucla.edu/), an audiovisual archive with an associated set of computational tools, hosted by the Library of the University of California Los Angeles and developed by the Red Hen Lab, an international consortium for research into multimodal communication (https://sites.google.com/site/distributedlittleredhen/). NewsScape offers streaming facilities of all the recorded audiovisual data, along with close captions corresponding to the transcription of the audio content. This results in a corpus of around 4 billion words occurring in over 250,000 hours of television news from 2004 to the present, mainly in English but also including smaller collections in at least twelve other languages [47]. Forced-alignment tools synchronize speech and subtitles with considerable precision, making it possible to search textual transcriptions and find the exact video moment in which a particular verbal pattern was uttered.

We utilized this resource to search for linguistic patterns in English corresponding to the four types of expressions that can be argued to typify the way that people talk about time across a wide range of cultures [37]:

*T-span*, *or demarcative temporal expressions* delimit a temporal process by signaling its starting point and/or its ending point, or by connecting two moments in time. We chose two very frequent and representative phrases in English: *from beginning to end* and *from start to finish*.

*Sequential expressions* situate two given events in a temporal sequence, specifying which event happens before or after another. There are many ways to indicate this in English; for our analysis, we again chose phrases that are frequently used as well as typical examples of this category: *earlier/later than* and *after/before that*.

#### Deictic directional expressions

These expressions locate time along the sagittal axis. The sagittal axis is verbalized in time expressions across languages, while the lateral is not [48]: “the past *behind* vs *on the left” or “the future *ahead* vs. *on the right”. Nevertheless, time deixis across cultures may make use of gesture along any axis, as well as of axis-independent gesture that signals a single point in space [49]. To examine pairs that are reasonably frequent and have clear opposites, we selected expressions containing the word *back*, such as “back in those days,” “back then,” or “back in 2001” and the word *ahead*, such as “days/months/years ahead,” or “time ahead.”

#### Deictic non-directional expressions

Finally, some expressions locate time in an undefined point in space. In some cases, such as in expressions of distance, the function is to locate the temporal unit or event in more or less proximity to the deictic center. To increase the variety in our sample with some more specific phrasal patterns, we selected these less broad but still frequent expressions: “distant past/future,” “far in the past/future,” and “near future”–“near past” has very low frequency.

Overall, this choice of expressions aimed at a combination of representativity, variety, symmetry, and frequency. We sought to include expressions that were unequivocally representative of their type. We also sought to showcase the variety of categories of time expressions and to include more than one expression per category. While doing this, we wanted to offer a balance in meaning between expressions of the same type, by including relevant pairs of opposites, such as *earlier-later* or *distant-near future* or, when clear opposites were not to be found, by including the arguably two most typical ways of instantiating the same time construction, as in *from beginning to end* and *from start to finish*. Alongside all this, we were looking for expressions frequent enough so that we could have comparable data across types, that is, we sought to avoid expressions rendering only a few hundred hits in the NewsScape repository.

Searches for each type of time expressions were kept within a range that was feasible for subsequent manual tagging. We adjusted the searches for each type of expressions until they rendered a number of hits between 1000–3000, and then we proceeded to filter out and annotate those clips. The scope of the search for time expressions simply sought to deal with the fact that the frequency of the expressions searched varies greatly in the NewsScape repository. For example, the expression “back in those days” appears 455 times in the whole NewsScape repository (from 2004 to 2017), while the expression “before that” appears 27,368 times. To achieve a balance between the types of time expressions studied, we manipulated the temporal scopes of the different searches so that we would achieve a comparable amount of hits for the different types of expressions (demarcatives, sequential, deictic directional, deictic non-directional), ranging from a minimum of 1000 hits and a maximum of 3000. This range allowed a sufficient number of cases for analysis, while also keeping the number of examples manageable for manual tagging. The smallest number of hits corresponded to non-directional deictic expressions (e.g., those containing the words “far”, “near”, “distant” or “close”), which are more specific and thus less frequent than the rest. This illustrates the sparsity problems inherent in linguistic data, and the corresponding requirement for very large datasets for quantitative studies such as the present one.

**Fig 1:**
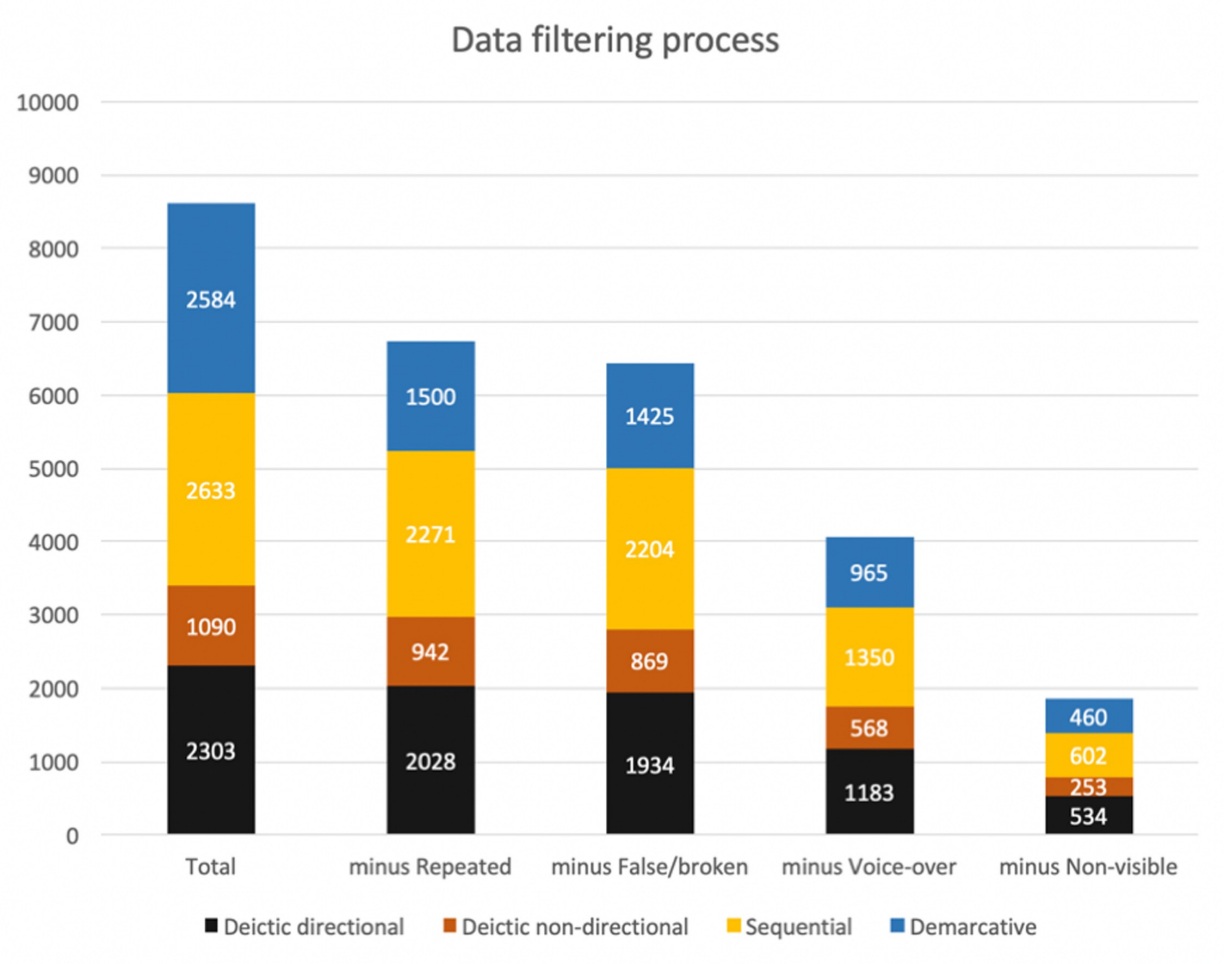
Data filtering from 8610 total hits to a corpus of 1849 clearly-visible clips.

This procedure allowed us to have comparable samples across all four types (see Fig 1). Sticking to such amounts and not seeking to obtain exactly the same number of clips for each expression or type were necessary measures for completing the data gathering process within a reasonable amount of time, given our capacities in that moment. Since the utterances studied were not tied to any particular historical events, speakers, or seasonal circumstances, considering their appearance over some months in a particular year or across several years does not affect the purposes of the present study.

A total sample of 8610 video clips containing utterances of these four types of time expressions was extracted for annotation. After the elimination of repeated, false/broken hits, voice-over or non-visible speaker instances, and clips where the hands of the speaker could not be clearly seen, this allowed for the production of a corpus of 1849 clearly-visible utterances which could be evaluated for the presence of gesture, along with its contribution to communication.

Searches for each expression yielded lists of clips corresponding to its appearances in the NewsScape video repository. Each clip was annotated by coders in several phases. In the first phase, coders filtered out clips that were repeated, presented technical problems, or rendered false hits. The NewsScape tools find all the instances in which a given phrase or word was uttered in a TV show included in the archive. This means that in some cases, e.g., interviews of public figures, international news, re-runs of a given recorded program or advertisements, the same clip could be shown in different channels at different times. These repetitions were duly noted and eliminated from the analyses. Other excluded clips were “broken links,” that is, very few clips that, for exceptional reasons, were technically flawed (e.g., the sound and the captions were not correctly aligned, or there was some problem with the sound or image of the clip). This segment also included the rare cases in which a hit did not correspond to the desired phrase. For example, when looking for the temporal expression “back then”, the system would sometimes find examples such as “He waved *back*. *Then*, he drove off”. In this example, the words “back” and “then” appear one after the other, but each is inserted in a different sentence. Those hits were also excluded. Overall, this first filtering phase eliminated around 18% of the initial hit list.

The remaining clips were then sorted between those that allowed a clear view of a speakers’ hands, and those in which the speakers’ hands could not be clearly seen. There were two main reasons for this: first, some clips were voice-overs, where the speaker does not appear in the clip, or situations in which we were seeing the speaker but the camera shot changed during the uttering of the expression searched. As a result, in those cases the voice uttering the expression could be heard, while the images showed something else. The second reason is that in some cases the speaker was shown but his/her hands were insufficiently visible or not visible at all, either due to the use of a close up or medium-close up take, in which only the head or the head and shoulders of the speaker are shown, or to the presence of visual obstacles such as captions, graphics, or other superimposed images. All clips in the first or second case were classified as ‘non-visible hands’ and filtered out.

A unique appearance per speaker was the typical case in the corpus, and only a few speakers, mostly news anchors and show hosts, appeared more than once. We annotated 100 random clips containing a time-related gesture for repeated speakers (sample also available at https://sites.google.com/site/creatimeproject/creatime-database) and found that 96% per cent of the clips contained unique speaker appearances. The remaining 4% contained repeated appearances of 3 different speakers (repeated speaker 1 appears 3 times, repeated speaker 2 appears twice, repeated speaker 3 appears twice). The sample contained all types of expressions, and speaker repetitions were not limited to a single individual expression or to a single type. While the number of repetitions could be slightly higher for the 923 clips containing relevant gesture and for the whole 8,610 clips in the database, with some of the 96% unique speakers in the sample re-appearing in some other clip, we can confidently say that the great majority of the clips analyzed contained an utterance by a unique speaker not appearing elsewhere in the data, and indeed almost never appearing elsewhere in the data for exactly the same temporal phrase. As a result, the impact of any repeated speaker or of the group of repeated speakers as a whole on the gesture frequency results for a particular expression or type was negligible in statistical terms.

A total of 75% of the hits were valid for the quantitative analysis, while the rest were repeated, broken, or false positives that were not actually examples of the phrases searched. The distribution of these figures did not vary substantially across the different types of temporal expressions. Valid hits were then further classified depending on the visibility of the hands of the speaker. In this phase, 38% of all valid clips were classified as containing a voice-over or non-visible speaker at the moment of utterance, 34% as “non-visible hands”, and the remaining 29% were the cases in which the hands of the speaker could be clearly seen. Again, as expected, the distribution of these categories did not vary much across the different types of temporal expression. The filtering process thus left us with 1849 clips to analyze for gesture, a little less than a third of all valid hits (29%). 2366 clips were classified as *voice-over* and 2217 as *non-visible hands* (see Fig 1).

To examine whether there was a statistical relation between the informativity of the time expressions searched and the rate of co-occurrence of their associated co-speech gesture, we used the frequency of the expressions in the COCA corpus as an indicator of their informativity. The COCA corpus contains more than 560 million words of text—20 million words each year 1990–2017, largely coinciding with the 2004–2017 dates of our searches in the NewsScape TV News Library. COCA is equally divided among spoken, fiction, popular magazines, newspapers, and academic texts. We chose the spoken portion of the COCA as the best proxy for the speech data gathered from NewsScape. That way our estimates of the frequency of the expressions searched were based on a spoken corpus that contained many other communicative settings besides television.

## Corpus annotation

**Fig 2:**
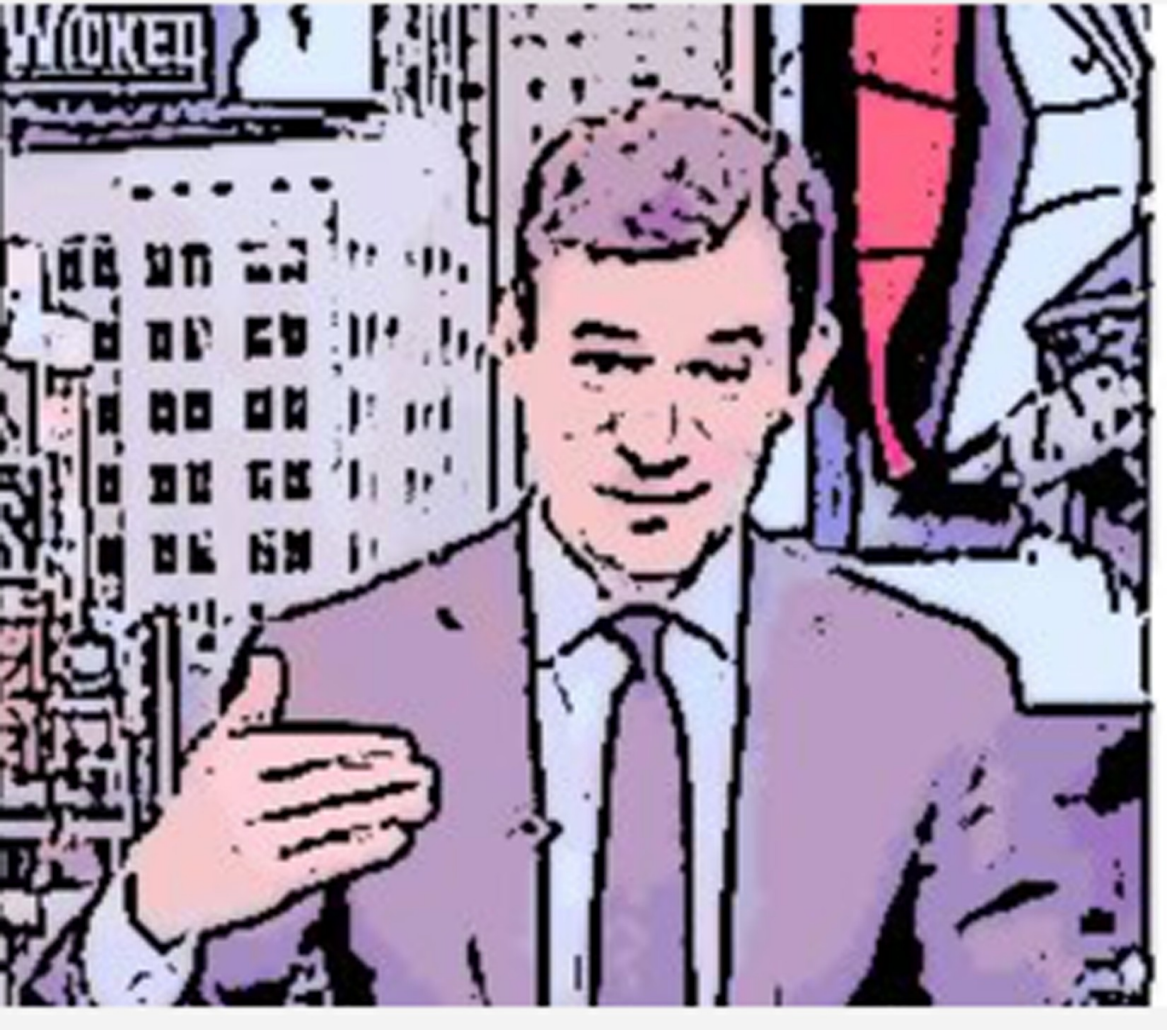
Illustration re-creating a real example of time-related gesture for “way distant future”. The speaker is extending his right arm towards the front, along a sagittal axis, simulating a path or timeline in which the future lies ahead.

The 1849 ‘clearly-visible’ clips were then classified into the three categories in Fig 1. These categories were distinguished according to the following criteria:

1. *No gesture was performed*. For the purposes of this study we restricted the scope of ‘gesture’ to what is also known as *gesticulation* [16]. Only the hand movements that accompany spoken language were targeted, and other bodily movements (head, facial expression, body posture) or extensions of the body (gaze) were not considered in these analyses.
2. *A gesture was performed and it was unrelated to time*. These gestures could not be related to the temporal meaning of the expression with total certainty. This was because they did not obviously cohere with the time expression (e.g. gestures that outlined no clear spatial pattern or could not be clearly paired with the linguistic expression because of lack of synchrony or any other discursive factor); because the gesture was clearly unrelated to time (e.g. raising thumb for approval, pointing at an element in the environment to refer to this element); or because the gesture, even if clearly paired with the expression and tracing a spatial pattern, was repeated throughout the preceding and/or subsequent discourse, indicating a discourse-segmentation or syntactic function (generally known as a *beat gesture* in gesture research). Therefore, this category includes gestures that may have not been signalling in coordination with the time expression, that may have had a semantic or pragmatic purpose unrelated to the expression of time, or that presented characteristics that were clearly incompatible with the formal features detected for temporal gestures in the literature [38–41]. Only gestures that were unambiguously connected to the expression of time were included in category 3.
3. *A gesture was performed and it was clearly related to the temporal meaning of the linguistic expression*. The clip contained a gesture that showed evidence of time spatialization, presenting formal features that were analogous to the gesture observed in previous research [38–41]. The speaker displaced one or both hands along one of the spatial axes to indicate durations, periods, or sequences, or pointed at a location in space corresponding to the moment in time expressed by the words (see Fig 2).

Each clip was annotated by two coders, all students at the University of Murcia and all of them naive to the study’s hypotheses. Annotation included a three-level confidence tag, ranging from 1 “Completely confident” to 2 “Almost sure” and 3 “Some doubts.” which helped locate problematic instances. We calculated inter-rater reliability in the different stages of the process. For the filtering-out process, no real disagreement was expected in the voice-over/non-visible speaker case or for the visibility of the hands. Our tests confirmed this expectation. A random selection of 200 clips showed a nearly perfect agreement between coders (N = 200, 99%; Cohen’s kappa = 0.98). We also checked the agreement in the distinction between clips with “no gesture” (hands can be clearly seen but no gesture is performed) and those clips containing some kind of gesture. Again, this distinction was not expected to be controversial and our intercoder reliability tests confirmed our assumption, with a nearly perfect agreement between coders on presence of gesture (N = 100, 95% agreement; Cohen’s kappa = 0.89).

The few disagreements between coders that did occur involved “no gesture” clips. Since we had instructed coders to annotate only clearly visible gestures, there was a small disagreement in the case of very small gestures, which were disregarded by one coder but not the other. In these cases, we adopted a conservative approach and the clip was in the end classified as “no gesture.” Finally, the most central classification for the purposes of this study, and arguably the most potentially sensitive, was the distinction between time-related vs non-time related gestures. In this case, we chose a bigger number of clips (N = 403) clips, which also showed a very high inter-coder agreement (92%); the Cohen’s kappa inter-agreement test scored 0.80, indicating a substantial agreement between the coders.

Therefore, the frequency rate of the time-related gesture co-occurring with these expressions in our corpus is, in all probability, higher than what our results reveal, since it is likely to have been depressed by our conservative filtering and annotation process. Although in some cases there was clearly no gesture, typical instances categorized as *non-visible hands* included clips in which the coders could indeed deduce that a gesture was being performed from observing the motion of shoulders or neck. However, whenever there was doubt, because the arms and hands did not appear on screen, or appeared too briefly, or could not be clearly seen for some other reason, clips were categorized as *non-visible*. Moreover, there were a number of instances, for both the *non-visible* and the *clearly-visible* categories, in which a time-related gesture was indicated by the speaker’s head and gaze, mainly using the lateral axis to situate temporal relations in a left-to-right or right-to-left timeline. We chose not to include those bodily expressions and to annotate hand gesture exclusively in order to further reduce interpretive bias.

The search results and the full annotations of the corpus used for this study are available from our CREATIME project website: https://sites.google.com/site/creatimeproject/creatime-database. That link also directs to the instructions for requesting access to the video collection from the Red Hen Lab directors. While all the metadata are in the file available from the CREATIME website, due to copyright restrictions we cannot provide access to the video clips. In compliance with the Copyright Law of the United States of America and Related Laws Contained in Title 17 of the United States Code (http://www.copyright.gov/title17/92chap1.html#108), § 108. Limitations on exclusive rights: Reproduction by libraries and archives, the UCLA NewsScape Library of Television News is construed to limit the reproduction and distribution by lending of a limited number of copies and excerpts of its audiovisual news program, subject to clauses 1, 2, and 3 of subsection (a) in the aforementioned law and title. Once permission to access the NewsScape Library has been obtained from the Red Hen Lab directors, we can provide the individual links to the video clips corresponding to each row in the metadata of the database used for the present study.

## Results

### Systematic gesture-speech co-occurrence

**Fig 3:**
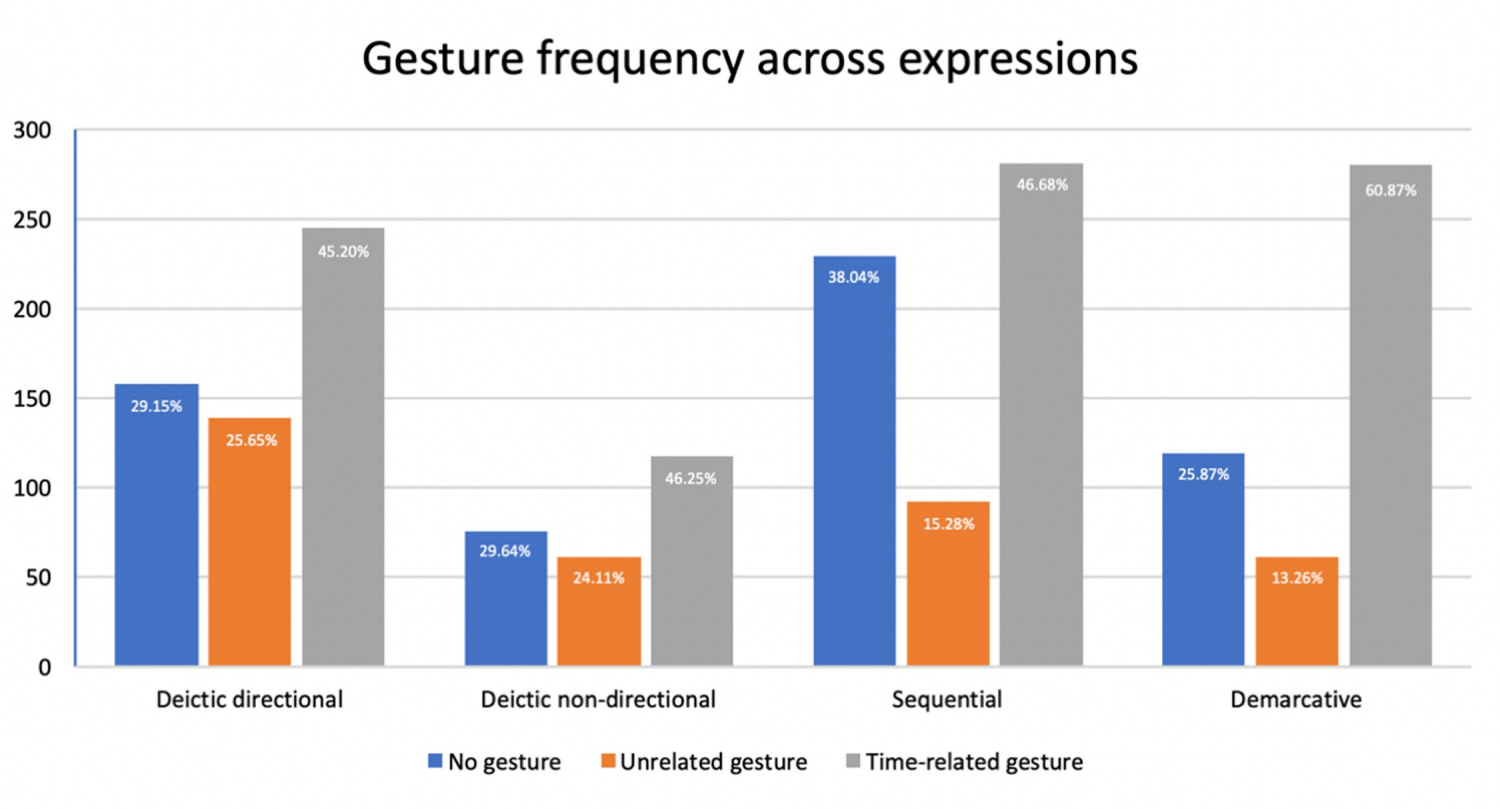
Gesture frequency across the different types of temporal expressions, with total number of clips and percentages.

Clearly-visible video clips were annotated for the presence or absence of gesture in relation with the time expression, with the following overall results (Fig 3):

1. No gesture was performed: 31% (581 clips out of a total of 1849 valid clips with the hands visible).
2. A gesture was performed that was unrelated to time: 19% (353 out of 1849).
3. A gesture was performed and it was clearly related to the temporal meaning of the linguistic expression, as evidenced by the use of gestural patterns such as those reported in the previous research (mainly signalling for a point ahead or behind the speaker or for an imaginary timeline along the lateral, sagittal, or vertical axis): 50% (923 out of 1849).

People gestured in 69% of the 1849 valid clips in which the time expressions searched were uttered and the hands were clearly visible, that is, in 1276 clips. Of these 1276 instances of co-speech gesture for demarcative, sequential, or deictic (directional or non-directional) time expressions, 72.33% were connected to time, while the rest were unrelated gestures, typically of the type known as *beat gestures*, signalling rhythm or segmentation for the ongoing discourse, or having some other discursive function.

Beyond this general breakdown, the distribution of gestures also differed among the different types of temporal expressions, as seen in Fig 3. Regarding the categories of temporal expressions examined here, speakers gestured less when making sequential expressions (*earlier/later than*), although even here the percentage of cases in which no gesture was produced was still a minority (38%). Of the remaining 62% of expressions where there was some gesture, 75% were related to time. Speakers in the two deictic categories of expression (directional and non-directional) showed a similar pattern of behavior, such that gestures were made in 70% of the clips in which these phrases were uttered, and around 65% of these gestures were time-related. Finally, demarcative expressions (*from beginning to end*) were accompanied by gesture in 74% of the samples examined (meaning that a mere 26% of the speakers examined did not gesture with their hands while uttering these expressions), with 82% of these gestures being related to time.

These data thus show that people tend to gesture when talking about time, and that time-related gestures co-occur frequently with all types of temporal expressions, adding a quantitative dimension to previous claims about the systematic relationship between speech and gesture. They also show that time-related gestures, even though always frequent, show considerable variation in their frequency of co-occurrence across specific verbal expressions. This variance could reflect differences in temporal meanings, as well as a range of contextual and situational factors that could have an influence on the gesture-speech relation: number of participants and their relative locations, the initial positions of their hands right before starting the gesture, whether they are holding objects in their hands or interacting with them in any other way, whether they are looking or attending to a screen or some other event, and so forth. None of these circumstances are particular to time expressions or to any specific type of them, and thus they can be expected to be randomly distributed throughout a sample of considerable size as the one used for the present study.

Accepting that all such circumstances influence co-speech gesture, we sought to look into a factor that could be related to the linguistic expressions themselves. The sub-corpus of speech and gestures aligned with a set of specific temporal phrases extracted above allowed us to make an objective, quantitative assessment of at least one of these factors possible. In order to explore whether the various modalities employed in communication contribute to an efficient system, we next examined the role that informativity plays in the relationship between gestures and the expressions they accompany. For this we took the frequency of each expression as a proxy to its predictability. Although, as explained in the introduction, predictability is often better accounted for by other behavioral measures [46], frequency correlates so strongly with these other factors that it is reasonable to take it as a proxy for them, given that frequency is much easier to quantify. Therefore, for present purposes, our hypothesis was that, all things being equal, less frequent expressions would be less predictable and therefore, if communication is working as a unitary system, this should increase the pressure to keep the levels of informativity of the communicative signal by adding information from other modalities. On the other hand, more frequent expressions would be generally more predictable, and then communicative efficiency should decrease the likelihood of a gesture co-occurring with them, given a dynamic system that seeks to minimize effort.

**Fig 4:**
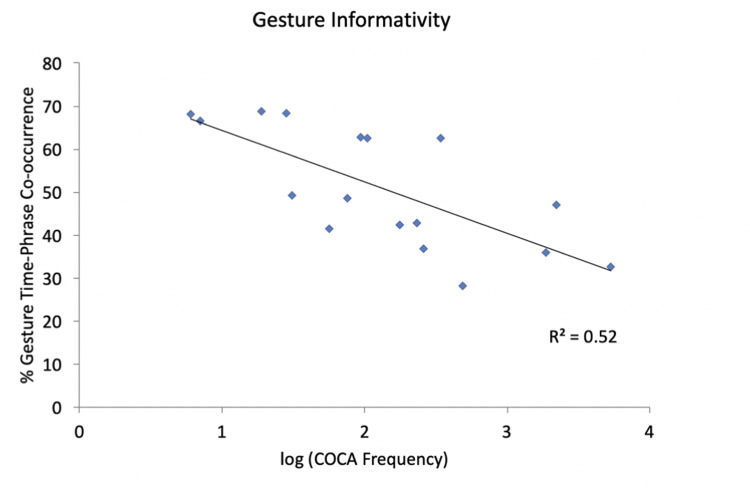
The spoken probability (message informativity) of the different types of temporal expressions (taken from COCA) plotted against the percentage of gestures co-occurring with each expression.

As Fig 4 shows, this analysis revealed a fairly close relationship between the predictability of a message (estimated from its frequency) and the likelihood of a gesture signal co-occurring with a verbal signal (R2 = .52). These data thus provide quantitative evidence of a relationship between the information provided by gestural signaling and the information provided by verbal signals. Moreover, in showing that speakers become more likely to make co-speech gestures as messages become less likely, the data indicate that co-speech gestures are efficient and integrated within a multimodal communicative system, because the overall effect of this pattern of co-occurrence will be to minimize the effort that speakers expend on gesture during communication, while at the same time maximizing the informativity of the integrated multimodal signal.

## Discussion

Even though all the data we analyzed were taken from television news programs in American English, the results reveal that temporal co-speech gestures are informative, efficient, highly systematic, and occur at high rates. While TV news shows cover a surprisingly wide range of communicative contexts (classic news reports delivered by a single news anchor facing the camera, multi-speaker interactions such as interviews, debates, talk shows, multi-speaker screenshots, and many more) television is often claimed to inhibit gesture [50], such that it follows that these data might actually represent an underestimate of the gesture rates in typical American English. Besides inhibiting gesture, television may also affect how gestures are performed. Politicians, professional broadcasters, and other public speakers often appear in our dataset, and many of them are trained to manage their body language in public speech, especially when they are facing cameras. To the best of our knowledge, we do not have any reliable data measuring how exactly the television environment affects gesture. However, we do know that the less naturalistic bodily expression that is common in television or in other forms of public speech is for the most part geared towards making gesture less frequent or less visible. Gesturing more and more ostensibly is not usually the goal of this training, and if some gestures are indeed rehearsed to be systematically performed or even enhanced at certain moments of discourse, they are likely to be related to key concepts or to discursive turns seeking certain effects, not to all-pervasive, multi-purpose time phrases. It is not easy to imagine a speaker training to perform expressions such as “earlier than” or “from beginning to end” in particular ways. Therefore, given the clarity of our findings, which is likely to be amplified beyond the newsroom, and the fact that American English is not usually regarded as an outlier in terms of manual expressivity, it can be expected that the overall patterns of behavior observed here will generalize to other languages.

Beyond these caveats, which include the fact that more data from languages other than American English are needed for comparison, the present study establishes, with a reliable quantitative power, a number of findings with considerable theoretical relevance. First, in a specific and very basic semantic domain such as time, conventional phrases representing the full gamut of variations in temporal meanings typically co-occur with a gesture that is related to that temporal meaning. This is supported by the overall rate of related gesture, which is 50% for such a conservative study as ours, as well as by the fact that for each category in our classification the related gesture rate was also close to 50% or higher. If, as a working hypothesis, we imagine an extrapolation of this finding to other semantic domains—for which we do not have data yet—we could consider the possibility that recurrent phrases in language are generally experienced, and therefore learned, alongside multimodal patterns—gestural and perhaps also involving other modalities as well—that cohere or coordinate with the verbal expression in some way. In any case, our findings strongly suggest that this is the case for the expression of time, and although some other domains may show different patterns, there is in principle no reason to suppose that time is a unique case.

The second major finding is that the need to discriminate between nuances in meaning can cause substantial differences in gesture frequency. In our corpus, demarcative phrases (“from beginning to end”) present a related gesture in almost two out of every three instances, while the other categories (sequential and deictic) have a related gesture rate slightly below 50%. This indicates that the frequency of co-speech gesture may contribute to signalling for semantic differences even within the same domain, by creating different expectations of the likelihood of gesture, based on statistical knowledge derived from exposure to usage. Such a finding calls for further, more detailed investigation into the relations between gesture frequency and meaning.

Finally, at the level of individual expressions and independently of any semantic classification, we have shown that co-speech gesture frequency of co-occurrence is a function of the predictability of the utterance, using linguistic frequency as a proxy. The less frequent a temporal phrase is, and therefore less predictable in general terms, the more likely it is to co-occur with a gesture related to the expression of time, and vice versa. This suggests that the communicative system is striving to keep the informativity of the signal at adequate levels, and that it seeks to achieve this with the maximum efficiency possible while minimizing effort.

Overall, the picture that emerges from these findings about co-speech gesture frequency and its contribution to efficiency in communication support the idea of a unitary complex dynamic system for communication. The systematicity of the relations between the different modalities seems to be very strong, and could perhaps go deeper and further than we might imagine. After all, what we have been presenting here are results about gesture frequency only. Some of the previous research using the NewsScape dataset, including our own, suggests that specific formal features of gesture may also be playing a role in the systematic differentiation of meaning and function, possibly also in the informativity of the signal. We look forward to further investigation triangulating all those factors with co-speech gesture frequency.

For those and other enquiries, we are confident that in the future we will be able to carry out much larger studies, thanks to the recent tools for the semi-automatic detection and annotation of verbal and non-verbal patterns that the Red Hen Lab has recently been developing for the NewsScape Library [51]. The goal of this study was to investigate levels of time spatialization and gesture-speech coupling in face-to-face communication using a large data set. While there are no clear parameters for what counts as a ‘large sample’ in this context, our methods, which sought to achieve the largest sample size possible in operational terms, yielded a corpus that clearly exceeds those used in previous psycholinguistic studies of gesture associated to any type of verbal patterns. As we describe in the introduction, previous studies typically considered samples of a few dozen gestures, not necessarily co-occurring with exactly the same verbal expressions and not necessarily related to a total number of utterances of those expressions. Alongise the few studies available that are based on Red Hen data, which we also referred to in the introduction, the present sample is arguably one of the largest ever used to study co-speech gesture associated to a specific set of linguistic expressions, and, to the best of our knowledge, the largest ever used for temporal phrases. It follows, of course, that when technical and operational means allow for a considerably larger sample size, we will be able to assess the relative value of the current sample as predictor for gesture frequency in real communicative settings. However, the soundness of the present approach is well supported by the clarity of the results it yields.

The data-driven, quantitative approach taken here is thus likely to yield fruitful results also with data from other modalities, thus contributing to a fuller picture of how human communication integrates its multiple channels/signals into an efficient whole. In combination with increasingly powerful tools such as those of the NewsScape Library, these methods will allow us to contrast results across representative corpora from different languages, or to quantify further aspects of cross-modal patterns with the help of technologies for the automatic detection of non-verbal signals, among many other exciting possibilities. This can lead to significant theoretical insights and subsequent practical applications across technologies based on models of human communication.
